# Randomized Clinical Trials of Deutaleglitazar in Healthy Participants and in Patients with Diabetic Kidney Disease

**DOI:** 10.1016/j.ekir.2026.107037

**Published:** 2026-08-20

**Authors:** Vlado Perkovic, Sam L. Francis, Hong Zhang, Ying Gao, Jicheng Lv, Haiyan Li, Yumei Yan, Yuran Zhang, Cathy Liu, Huading Zhang, Jin Tian

**Affiliations:** 1 University of New South Wales, Sydney, Australia; 2 Nucleus Network Pty. Ltd, Melbourne, Australia; 3 Peking University First Hospital, Beijing, China; 4 Peking University Third Hospital, Beijing, China; 5 Alebund Pharmaceuticals, Shanghai, China

**Keywords:** deutaleglitazar, diabetic kidney disease, GFR, HDL-C, pharmacokinetics, PPAR agonist

## Abstract

**Introduction:**

Deutaleglitazar (AP303) is a novel dual peroxisome proliferator-activated receptor (PPAR) α and γ agonist, which has the potential to offer the combined clinical benefit on diabetic kidney disease (DKD) management by normalizing the elevated glomerular capillary pressure, ameliorating podocyte depletion, as well as correcting diabetic dyslipidemia and hyperglycemia

**Methods:**

A total of 3 single-center, randomized, placebo-controlled single-ascending-dose and/or multiple-ascending-dose studies investigated its pharmacokinetics (PK), pharmacodynamic, safety, and tolerability in healthy participants with different ethnicities and in patients with DKD and reduced kidney function.

**Results:**

A total of 80 healthy participants, and 18 patients with DKD and estimated glomerular filtration rate (eGFR) 30–60 ml/min per 1.73 m^2^ from Australia and China received either placebo, a single oral dose, or multi-dose of deutaleglitazar for 14 days. Deutaleglitazar exposure increased in a dose-dependent manner both after a single dose and at steady state, with no accumulation. Minor differences of PK profiles in Caucasian versus Asian participants, and in those with normal versus reduced kidney function are considered unlikely to be clinically significant. Reduction in eGFR with reversibility after drug discontinuation was evident. Improvement in diabetic dyslipidemia and hyperglycemia were observed. Few adverse events were reported; only neutropenia was dose related.

**Conclusions:**

The PPARα and PPARγ related effects occurred over similar dose ranges, indicating that deutaleglitazar is a balanced agonist of the 2 receptor subtypes targeting on the root cause and multi-pathway of DKD progression.

## Graphical abstract

**Figure:**
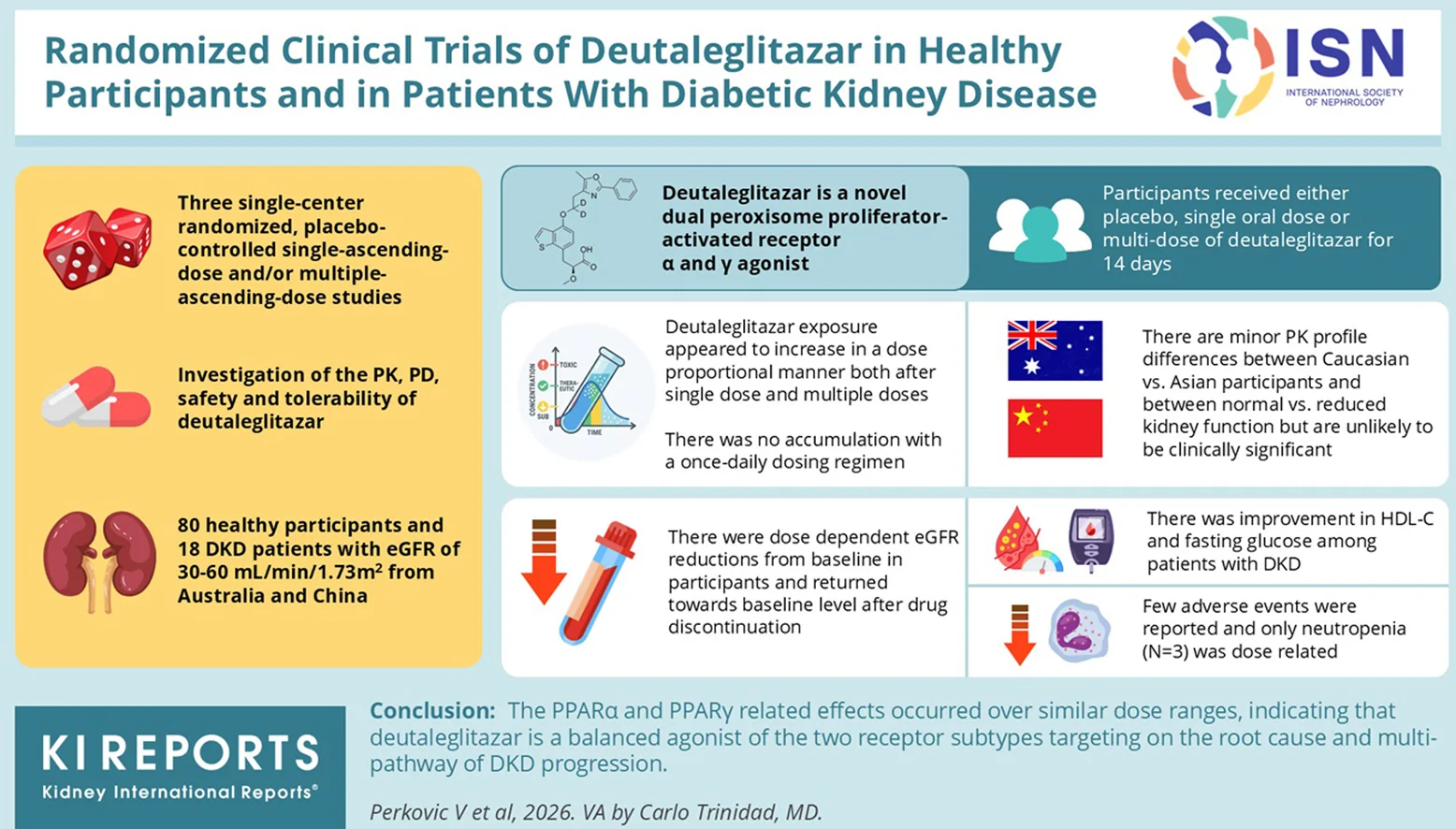

DKD is a chronic, progressive condition mediated through glucose-induced injury to the renal microvasculature. Most cases occur in patients with type 2 diabetes (T2D), where insulin resistance and dyslipidemia play important roles in its pathogenesis. In 2021, 537 million people worldwide (11% of the global population) had diabetes, and this number is expected to increase to 783 million by 2045.^1^ About 25%−40% of T2D patients will develop DKD within 20 years after the initial diagnosis.^2^ The clinical presentation of DKD includes persistent albuminuria and early elevation of the GFR followed by the progressive decline in renal function. However, mortality from cardiovascular disease commonly occurs before DKD has progressed to kidney failure.^3^

Hemodynamically mediated glomerular injury leading to a compensatory increase in single-nephron glomerular filtration rate has been widely accepted as the theory for DKD progression.^4^ Even though this initially compensates for kidney function, it ultimately leads to glomerular hypertrophy and podocyte damage, thereby perpetuating a vicious cycle of nephron loss. Podocytes are particularly subjected to mechanical stress under hyperfiltration that triggers inflammation, podocyte injury, and depletion, which present clinically as increased albuminuria. Aiming to reduce the glomerular capillary pressure (presenting clinically as the initial drop of eGFR) either by dilation of efferent arteriole (renin-angiotensin system blockade) or constriction of afferent arteriole (sodium-glucose cotransporter 2 inhibitor) is a key mechanism to attenuate the functional progression of DKD.^5^^,^^6^ However, because of the ceiling effect of renin-angiotensin system blockade and sodium-glucose cotransporter 2 inhibitor on glomerular hemodynamic changes,^7^ they can only slow down the progressive loss of kidney function but are unable to correct the intraglomerular pressure to the normal physiologic level. Even though glomerular hyperfiltration is dominant and may trigger podocyte depletion, certain products may decrease proteinuria independent of glomerular hemodynamic changes (without a clear or only had minimal initial decline in eGFR), such as glucagon-like peptide-1 agonists.^8^ As such, it may be that glomerular hyperfiltration and podocyte depletion are dual drivers of glomerular injury and DKD progression.^9^

Peroxisome proliferator activated receptors are ligand dependent transcription factors that control gene expression. Both PPARα (e.g., fenofibrate) and PPARγ agonists (e.g., pioglitazone) have been widely used clinically in patients with T2D for the improvement of dyslipidemia or for the improvement of glycemic control and insulin resistance respectively. Interestingly, certain PPARα agonists showed a clear glomerular hemodynamic effect suggesting a reduction in intraglomerular pressure. The Fenofibrate Intervention and Event Lowering in Diabetes study evaluated the effect of fenofibrate on cardiovascular events in T2D patients. In this study, a mean increase in serum creatinine levels of 12% from baseline was observed in the fenofibrate group. This effect persisted over 5 years, returning to below-placebo levels over the 8-week period after fenofibrate was withdrawn.^10^ In addition, treatment with the PPARγ agonist pioglitazone resulted in a significant albuminuria reduction as well as significantly reduced urinary podocyte excretion in patients with DKD.^11^ Because the reduction of urinary podocyte and albuminuria was not associated with the decreased GFR, PPARγ agonist could exert a direct effect on podocyte protection. Deutaleglitazar (AP303) is a novel, balanced, dual PPARα, and PPARγ agonist. We conducted 3 placebo-controlled randomized studies to investigate the PK, pharmacodynamic, safety, and tolerability of deutaleglitazar in healthy participants from different ethnicities and in patients with DKD and impaired kidney function.

## Methods

### Study Design

The first-in-human study (AP303-PK-01, ClinicalTrials.gov identifier NCT05503693) was a single-center, randomized, double-blind, placebo-controlled, single-ascending-dose (SAD), and multiple-ascending-dose (MAD) study in healthy participants, conducted in Australia. Qualified participants were randomly assigned to a single dose of deutaleglitazar (50, 150, 300, and 600 μg) or a placebo in the SAD cohorts. In the Food effect cohort, participants received deutaleglitazar 150 μg under fasting conditions at Day 1, and under a high-fat high-calorie meal after 14 days of washout. In the MAD cohorts, participants received daily dose of deutaleglitazar 50, 150, and 300 μg for 14 days. Treatment was formulated as tablets of deutaleglitazar at 2 dose strengths (50 μg and 150 μg) or matching placebo tablets. Higher dose levels were initiated only after evaluation of the tolerability, safety, and the PK data 24 hours after the last dose of the lower dose cohort. AP303-PK-02 (ClinicalTrials.gov identifier NCT06308523) was a single-center, randomized, double-blind, placebo-controlled multiple-ascending-dose phase 1 study in healthy Chinese participants. The phase 1b study (AP303-PK-03, ClinicalTrials.gov identifier NCT06666283) was a single-center, randomized, double-blind, placebo-controlled multiple-dose study in Chinese patients with DKD and renal impairment (eGFR 30-60 ml/min per 1.73 m^2^). On the basis of the data from the study AP303-PK-01, once-a-day oral administration of deutaleglitazar 150 μg or 300 μg for 14 days was selected in these 2 multi-dose studies. In all 3 studies, before the start of the study, a randomization schedule was created by an unblinded statistician from an independent vendor, and code break envelopes were available for emergency unblinding. In AP303-PK-01, as a conservative approach for this first in human study, each cohort except for SAD Cohort 2 (food effect cohort), randomization to AP303 or placebo was in 6:2 ratio (Sentinel 1:1, rest of cohort 5:1), in SAD Cohort 2, randomization to AP303 or placebo was in 12:2 ratio (Sentinel 1:1, rest of cohort 11:1); in MAD cohorts, randomization to AP303 or placebo was in 6:2 ratio, 62 participants in total. In AP303-PK-02 and AP303-PK-03, randomization to AP303 or placebo was in 2:1 ratio, 9 participants were randomized with 6 participants to receive AP303 and 3 participants to receive matching placebo for each cohort, 18 participants in total for each study.

In study AP303-PK-01, participants stayed at the study center from the evening of day-1 (1 day before the commencement of the treatment) to the morning of day 3 after the last dose (4 days for SAD cohorts and 17 days for MAD cohorts). Each dose was taken with 240 mL of water. Blood samples for intensive PK assessments were collected at pre dose, 0.5, 1, 2, 3, 4, 6, 8, 10, 12, 24, 48, 72, and 96 hours after dose at day 1, MAD day 1 (except 48–96 hours) and MAD day 14. Trough (pre dose) samples were collected on days 2, 3, 4, 5, 7, 12, and 13 in MAD cohorts. In study AP303-PK-02, blood samples for PK assessments were collected at pre dose, 0.5, 1, 2, 3, 4, 6, 8, 10,12, 24, and 48 hours after the first dose (day 1) and last dose (day 14). Trough samples were collected on Days 6, 7, 10, and 14 before the dose (i.e., pre dose). In phase 1b study (AP303-PK-03), blood samples for PK assessments were collected at pre dose, 0.25, 0.5, 1, 2, 3, 4, 6, 8, 10, 12, 24, and 48 hours after dose at day 1 and day 14. Trough (pre dose) samples were collected on days 4, 6, 10, and 14.

The PK parameters of deutaleglitazar included the area under the plasma concentration–time curve (AUC_0-∞_, AUC_0-last_, AUC_0-24h_, AUC_0-tau_), maximum plasma concentration (C_max_), trough plasma concentration (C_min_), mean plasma concentration (AUC_0-24h_/24, C_av_), time to C_max_ (T_max_), t_½_, apparent clearance of the orally administered drug (CL/F), apparent volume of distribution of the orally administered drug (V/F). Accumulation index (Rac) was calculated on days 1 and 14, using standard noncompartmental methods. Urine samples were collected for PK analysis in the first-in human study.

The pharmacodynamic assessments in study AP303-PK-01 MAD cohort, AP303-PK-02, and AP303-PK-03 studies included the changes in metabolic profile [fasting glucose, total cholesterol (TC), triglyceride (TG), high-density lipoprotein cholesterol (HDL-C), and low-density lipoprotein cholesterol (LDL-C)], and the change in kidney function (serum creatinine and eGFR) from baseline to the end of 14 days of treatment and 14 days after the last dose. The safety and tolerability assessments, including vitals, body weight, physical examination, EKG, hematology, blood biochemistry, coagulation, troponin I, and urinalysis, were performed following the schedule of assessment in the protocol.

### Participants

All participants provided their written informed consent, and the study protocols were approved by the local ethics committees. All the studies were conducted in accordance with the provisions of the Declaration of Helsinki and Good Clinical Practice guidelines. In study AP303-PK-01, healthy male and female (aged 18–55 years) were recruited from 1 center in Australia. In study AP303-PK-02, healthy male and female (aged 18–50 years) were recruited from 1 center in China. In study AP303-PK-03, male and female patients with DKD (age ≥ 30 years) receiving at least 1 glucose lowering product and on stable dose of angiotensin-converting enzyme inhibitor or angiotensin II receptor blocker for at least 4 weeks were recruited from one center in China. To be eligible for AP303-PK-03, patients were also required to have body mass index 18∼30 kg/m^2^, HbA1c 6.5%–10.5%, eGFR 30–60 mL/min per 1.73 m^2^ and urinary albumin-to-creatinine ratio (UACR) ≥ 30 mg/g at screening. Participants were not eligible if they were receiving any PPAR agonist, or with an ALT or AST > 1.5 × upper limit of normal, or any other clinically significant abnormalities in liver function test at screening.

### Statistical Analysis

Sample size was selected to provide sufficient data characterizing PK profile (6 participants in deutaleglitazar arm, 2 or 3 in placebo arm) for each dose level in each study. The safety analysis set is defined as all participants who have received at least 1 dose of the study drug, the PK analysis set includes all participants who receive at least 1 dose of the study drug and have at least one evaluable PK measurement, and the pharmacodynamics analysis set (PDS) includes all participants who receive at least one dose of the study drug and have at least one evaluable pharmacodynamic measurement. The PK, pharmacodynamic, and safety data were all summarized using descriptive statistics based on PK analysis set, pharmacodynamics analysis set, and safety analysis set, respectively. No missing data were imputed.

## Results

### Patient Disposition and Demographics

A total of 62 healthy participants from Australia were randomized and dosed in study AP303-PK-01, 18 healthy participants from China in AP303-PK-02, and 18 patients with DKD and renal impairment in study AP303-PK-03, and all their data were included in the safety analysis set. A total of 2 participants (psychologic symptoms, neutropenia) in AP303-PK-01 and 2 participants (pain, increase in serum creatinine) in AP303-PK-03 were early terminated from study. In AP303-PK-03 study, 100% of patients received angiotensin-converting enzyme inhibitor or angiotensin II receptor blocker, 44% received 2 or 3 approved medications for DKD including angiotensin-converting enzyme inhibitor or angiotensin II receptor blocker, SGLT2i, GLP1RA, or MRA; 100% received at least 1 glucose lowering product, 56% received 2 or more medications for glycemic control. The demographic characteristics and baseline PD parameters are listed in Table 1.

**Table 1:** Demographic characteristics and baseline PK parameters for all 3 studies

Characteristic / PD parameter	PK-01									PK-02			PK-03	
	SAD				MAD					SAD+MAD			Multi-Dose	
	Placebo	50 μg	150 μg	300 μg	600 μg	Placebo	50 μg	150 μg	300 μg	Placebo	150 μg	300 μg	Placebo	150 μg
*n*	8	6	12	6	6	6	6	6	6	6	6	6	6	12
Ages (y)	30.3 ± 5.90	28.7 ± 12.86	28.8 ± 8.79	28.2 ± 9.06	27.7 ± 9.56	29.5 ± 6.19	27.3 ± 5.20	32.8 ± 8.01	30.7 ± 8.76	31.2 ± 8.86	28.0 ± 4.29	31.2 ± 4.54	59.0 ± 11.22	64.3 ± 8.42
Male^a^, *n* (%)	4 (50.0)	4 (66.7)	8 (66.7)	3 (50.0)	1 (16.7)	4 (66.7)	5 (83.3)	5 (83.3)	2 (33.3)	3 (50.0)	5 (83.3)	2 (33.3)	4 (66.7)	10 (83.3)
Race, *n* (%)														
American Indian or Alaska Native	0 (0)	0 (0)	0 (0)	0 (0)	0 (0)	0 (0)	0 (0)	0 (0)	1 (16.7)	0 (0)	0 (0)	0 (0)	0 (0)	0 (0)
White	5 (62.5)	4 (66.7)	10 (83.3)	2 (33.3)	6 (100)	4 (66.7)	6 (100)	4 (66.7)	4 (66.7)	0 (0)	0 (0)	0 (0)	0 (0)	0 (0)
Asian	3 (37.5)	2 (33.3)	2 (16.7)	4 (66.7)	0 (0)	1 (16.7)	0 (0)	2 (33.3)	1 (16.7)	6 (100)	6 (100)	6 (100)	6 (100)	12 (100)
Multiple	0 (0)	0 (0)	0 (0)	0 (0)	0 (0)	1 (16.7)	0 (0)	0 (0)	0 (0)	0 (0)	0 (0)	0 (0)	0 (0)	0 (0)
Weight (kg)	68.34 ± 7.98	79.07 ± 9.56	73.76 ± 16.73	66.77 ± 6.79	66.67 ± 20.68	70.10 ± 16.56	70.17 ± 11.06	78.60 ± 13.58	72.50 ± 9.34	65.02 ± 7.78	69.72 ± 7.74	60.17 ± 6.59	68.92 ± 9.73	77.53 ± 10.44
Height (cm)	170.4 ± 11.78	173.5 ± 9.89	174.2 ± 13.14	170.0 ± 7.80	168.2 ± 18.67	167.5 ± 9.67	172.7 ± 9.16	178.5 ± 11.55	166.7 ± 7.58	163.25 ± 9.82	171.08 ± 10.19	165.50 ± 8.40	163.27 ± 10.22	168.43 ± 8.33
BMI (kg/m^2^)	23.64 ± 2.56	26.25 ± 1.98	24.04 ± 3.65	23.13 ± 2.51	23.20 ± 3.06	24.73 ± 4.20	23.47 ± 2.90	24.63 ± 3.25	26.22 ± 3.99	24.27 ± 1.28	24.02 ± 0.56	22.13 ± 1.73	25.76 ± 2.43	27.27 ± 2.25
FPG (mmol/L)	4.53 ± 0.18	4.53 ± 0.30	4.33 ± 0.35	4.32 ± 0.52	4.42 ± 0.37	4.50 ± 0.50	4.50 ± 0.34	4.63 ± 0.20	4.33 ± 0.15	4.88 ± 0.18	4.95 ± 0.23	5.05 ± 0.32	7.55 ± 1.70	7.53 ± 1.74
HbA1c (%)	-	-	-	-	-	-	-	-	-	-	-	-	7.72 ± 1.07	7.83 ± 0.89
TC (mmol/L)	-	-	-	-	-	4.82 ± 0.94	3.98 ± 0.39	4.35 ± 0.40	5.02 ± 0.92	4.31 ± 0.38	4.47 ± 0.76	4.41 ± 0.79	4.18 ± 1.41	4.49 ± 0.98
LDL-C (mmol/L)	-	-	-	-	-	2.88 ± 0.73	2.15 ± 0.55	2.67 ± 0.38	2.90 ± 0.69	2.72 ± 0.35	2.77 ± 0.56	2.44 ± 0.70	2.13 ± 0.89	2.57 ± 0.76
HDL-C (mmol/L)	-	-	-	-	-	1.48 ± 0.33	1.43 ± 0.39	1.12 ± 0.31	1.82 ± 0.26	1.25 ± 0.25	1.35 ± 0.22	1.52 ± 0.19	1.02 ± 0.18	0.95 ± 0.23
TG (mmol/L)	-	-	-	-	-	0.95 ± 0.77	0.87 ± 0.43	1.22 ± 0.86	0.67 ± 0.21	1.32 ± 1.04	1.35 ± 0.58	0.86 ± 0.30	2.13 ± 1.07	2.02 ± 0.93
eGFR (ml/min 1.73 m^2^)	-	-	-	-	-	95.5 ± 10.39	89.5 ± 14.88	91.5 ± 8.22	93.7 ± 9.79	121.2 ± 8.28	108.8 ± 9.89	122.7 ± 4.41	49.1 ± 5.62	45.2 ± 6.94
UACR (mg/g)													1328.1	1185.6
Products for DKD (%)														
ACEi/ARB	-	-	-	-	-	-	-	-	-	-	-	-	100	100
≥ 2 meds	-	-	-	-	-	-	-	-	-	-	-	-	33.3	50
Glucose lowering product (%)														
1	-	-	-	-	-	-	-	-	-	-	-	-	50.0	41.7
2	-	-	-	-	-	-	-	-	-	-	-	-	16.7	58.3
≥ 3	-	-	-	-	-	-	-	-	-	-	-	-	33.3	0

ACEi, angiotensin-converting enzyme inhibitor; ARB, angiotensin II receptor blocker; BMI, body mass index; DKD, diabetic kidney disease; eGFR, estimated glomerular filtration rate; FPG, fasting plasma glucose; HbA1c, hemoglobin A1c; HDL-C, high-density lipoprotein cholesterol; LDL-C, low-density lipoprotein cholesterol; MAD, multiple-ascending-dose; meds, medications; PD, pharmacodynamic; SAD, single-ascending-dose; TC, total cholesterol; TG, total triglyceride. UACR, urinary albumin to creatinine ratio.

a Sex is specified as collected at birth.

### Pharmacokinetics

#### PK Parameters

**Figure 1:**
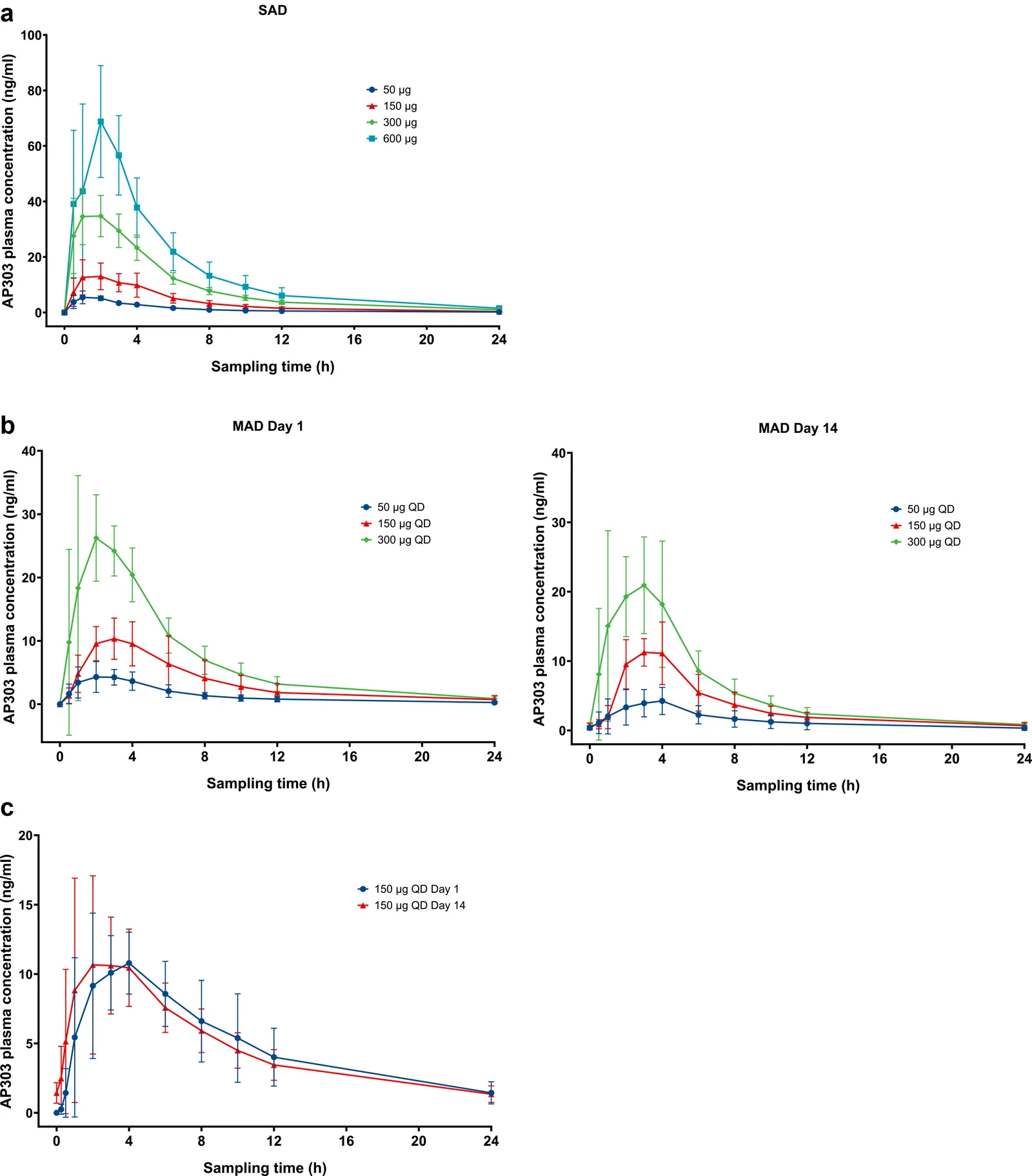
Mean (± SD) plasma concentration-time profile of AP303. (a) AP303-PK-01 SAD cohorts in heathy participants; 50 μg, 150 μg, 300 μg, and 600 μg; (b) AP303-PK-01 MAD cohorts in healthy participants on day 1 after a single dose and day 14 after multiple doses; 150 μg, 150 μg, 150 μg; (c) AP303-PK-03 150 μg QD day 1 after a single dose and day 14 after multiple doses in patients with DKD and eGFR 30–60 ml/min per 1.73 m^2^. eGFR, estimated glomerular filtration rate; MAD, multiple-ascending-dose; SAD, single-ascending-dose; QD, once daily.

The mean plasma concentration-time profile of deutaleglitazar after drug administration is shown in Figure 1. The peak concentration of deutaleglitazar was observed at median T_max_ ranging from 0.5 to 4 hours, and the estimates of the mean half-life were in the range of 5 to 11 hours. Deutaleglitazar exposure appeared to increase in a dose proportional manner both after single dose and multiple doses (Figure 1a and b). Steady trough concentrations (C_trough_) of deutaleglitazar indicated steady state could be attained by day 2 with repeat daily dosing. There was no accumulation with a once-daily dosing regimen. A high-fat meal did not affect overall bioavailability (Table 2). Unchanged parent drug was minimally excreted in the urine, and was below the limit of quantification in majority of participants (45/48).

**Table 2:** Summary of PK parameters of 150 μg AP303 from all 3 studies

PK parameter	AP303-PK-01				AP303-PK-02		AP303-PK-03	
	Fasting	Fed	Day 1	Day 14	Day 1	Day 14	Day 1	Day 14
AUC_0-inf_ (ng∗h/mL)	85 ± 23.5	88 ± 34.4	87 ± 41.5	89 ± 32.5	108 ± 22.2	107 ± 21.1	135 ± 41.4	136 ± 39.3
AUC_0-24_ (ng∗h/ml)	-	-	80 ± 34.5	-	99 ± 18.5	-	114 ± 30.6	-
AUC_0-last_ (ng∗h/ml)	82 ± 22.7	86 ± 34.3	80 ± 34.2	86 ± 32.5	106 ± 22.1	104 ± 21.4	127 ± 40.2	130 ± 35.3
AUC_0-tau_ (ng∗h/ml)	-	-	-	78 ± 25.0	-	96 ± 17.6	-	112 ± 27.1
C_max_ (ng/ml)	16.3 ± 4.07	12.0 ± 4.51	12.5 ± 2.56	13.4 ± 3.74	22.4 ± 4.53	23.7 ± 3.45	13.1 ± 2.62	14.6 ± 4.80
T_max_^a^ (h)	2.0 (0.5–4.03)	2.2 (1–6.72)	3.0 (2–6)	3.0 (2–4)	0.5 (0.5–0.5)	0.5 (0.5–0.5)	3.5 (1–10)	2.0 (1–8)
t_½_ (h)	5.2 ± 0.52	6.6 ± 2.05	6.7 ± 0.94	9.2 ± 2.98	8.2 ± 1.51	9.7 ± 1.94	9.3 ± 1.67	10.9 ± 1.88
C_av_ (ng/mL)	-	-	-	3.23 ± 1.04	-	4.00 ± 0.73	-	4.66 ± 1.13
C_min_ (ng/ml)	-	-	-	-	-	0.62 ± 0.24	-	1.25 ± 0.65
CL/F (l/h)	1.92 ± 0.64	1.94 ± 0.77	2.01 ± 0.77	2.11 ± 0.68	1.45 ± 0.36	1.62 ± 0.38	1.20 ± 0.33	1.41 ± 0.34
V/F (l)	14.2 ± 4.62	17.0 ± 3.89	18.6 ± 5.36	26.1 ± 4.77	16.5 ±1.97	22.3 ± 4.58	15.9 ± 4.58	21.8 ± 4.99
R_ac_ AUC	-	-	-	1.00 ± 0.14	-	0.97 ± 0.09	-	1.00 ± 0.20
R_ac_ C_max_	-	-	-	1.08 ± 0.25	-	1.10 ± 0.30	-	1.14 ± 0.30

AUC_0-inf_, area under the plasma concentration versus time curve extrapolated to infinity; AUC_0-last_, area under the plasma concentration versus time curve up to the last measurable concentration; AUC_0-24h_, area under the plasma concentration versus time curve up to 24 hours; AUC_0-tau_, area under the plasma concentration versus time curve for a dosing interval; C_av_, average plasma concentration; C_max_, maximum observed plasma concentration; CL/F, apparent oral clearance calculated from dose/AUC_0-tau_; C_min_, minimum observed plasma concentration; PK, pharmacokinetic; R_ac_, ratio of accumulation; T_max_, time to maximum observed plasma concentration; t_1/2_, apparent terminal half-life; V/F, apparent volume of distribution of oral drug.

Baseline eGFR was 91.5 ± 8.22 ml/min per 1.73 m^2^ in AP303-PK-01 150 μg group in MAD cohort; 108.8 ± 9.89 ml/min per 1.73 m^2^ in AP303-PK-02 150 μg group; and 45.2 ± 6.94 ml/min per 1.73 m^2^ in AP303-PK-03 150 μg group.

a Median [min-max] is presented for T_max_.

#### Pharmacokinetic Parameters in Different Ethnicity

In study AP303-PK-01, the extent of absorption of deutaleglitazar appeared to be higher in Asian participants compared with non-Asian participants. In comparison with healthy Australian participants (AP303-PK-01) at the same dose, the AUC_0-inf_ and C_max_ of healthy Chinese participants (AP303-PK-02) were also higher than those of healthy Australian participants, and this difference may be related to weight differences. Using SAD PK data from AP303-PK-01 and AP303-PK-02, analyzing with model log (PK ∗ weight/dose)∼race, the 90% confidence interval of Asian or non-Asian for all PK end points is very close to the equivalent threshold (0.8, 1.25), and no ethnic differences were found. Detailed results are presented in Table 3.

**Table 3:** Analysis of dose- and body weight-adjusted AP303 PK parameters [Model log (PK ∗ weight/dose) ∼race] in Asian versus Non-Asian participants across all PK end points (Studies AP303-PK-01 and AP303-PK-02)

PK parameter	Number of participants Asian / Non-Asian	Geometric mean (Asian)	Geometric mean (Non-Asian)	Geometric mean Ratio estimate (Asian/Non-Asian)	90% CI estimate (Asian/Non-Asian)
AUC_0-inf_	20 / 22	45.47 ng∗h/ml	41.26 ng∗h/ml	1.10	(0.97, 1.26)
AUC_0-last_	20 / 22	44.46 ng∗h/ml	40.10 ng∗h/ml	1.11	(0.97, 1.26)
C_max_	20 / 22	9.13 ng/ml	7.93 ng/ml	1.15	(1.03, 1.29)

AUC_0-inf_, area under the plasma concentration versus time curve extrapolated to infinity; AUC_0-last_, area under the plasma concentration versus time curve up to the last measurable concentration; CI, confidence interval; C_max_, maximum observed plasma concentration; PK, pharmacokinetic.

The average body weight for Asian was 66.40 kg, for non-Asian was 73.25 kg.

#### PK Parameters in Patients With Impaired Kidney Function

Patients with DKD and impaired renal function are the target population of deutaleglitazar, the effect of kidney function on PK of deutaleglitazar was investigated in this population in study AP303-PK-03. After the completion of cohort 1 (150 μg QD [once daily]) in AP303-PK-03, the safety review committee recommended not escalating to 300 μg QD but to keep 150 μg QD for patients in cohort 2 to fulfill the US Food and Drug Administration minimal sample size requirement for renal impairment study based on the higher variability of PK parameters as well as the safety data especially the magnitude of eGFR changes from baseline in cohort 1. The mean plasma concentration-time profiles of deutaleglitazar after 150 μg administration on days 1 and 14 are shown in Figure 1c. There was no accumulation with a once-daily dosing regimen. The PK parameters of 150 μg cross all 3 studies under different mean eGFR are listed in Table 2. The absorption of deutaleglitazar was delayed and the mean C_max_ was reduced (by 38.4%) but the AUC_0-tau_ was mildly increased (by 16.7%) in patients with renal impairment (AP303-PK-03) compared with normal kidney function in participants with the same ethnic background (AP303-PK-02).

### Pharmacodynamics

#### eGFR Change

**Figure 2:**
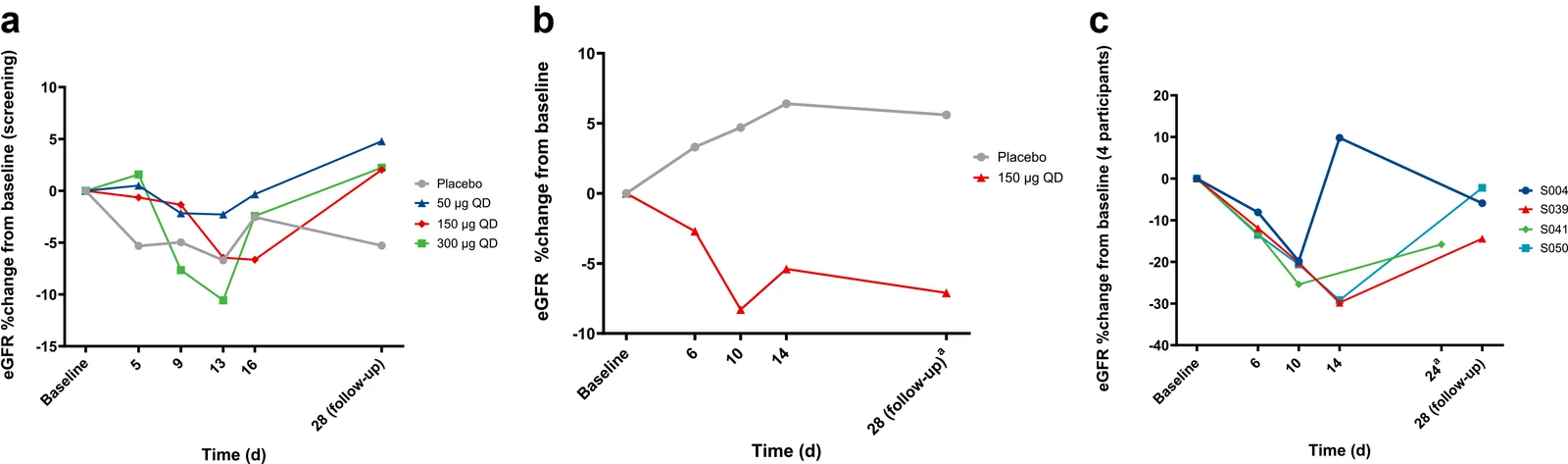
eGFR changes in healthy participants with normal kidney function and in patients with DKD and renal impairment (eGFR 30–60 ml/min per 1.73 m^2^). (a) Mean eGFR changes in healthy participants with normal kidney function (data from AP303-PK-01 study); (b) Mean eGFR changes in patients with DKD and eGFR 30–60 ml/min per 1.73 m^2^ (data from AP303-PK-03 study); (c) eGFR % change from baseline in individual patients with eGFR reduction ≥ 20 % from baseline during treatment in patients with DKD (data from AP303-PK-03 study). eGFR, estimated glomerular filtration rate; QD, once daily. ^a^Participant S041 discontinued treatment early after the last dose on day 10. Consequently, the follow-up visit was conducted on day 24 (c), and the eGFR value obtained at this visit was included in the day 28 (follow-up) analysis window (b).

In study AP303-PK-01 MAD cohorts, the reduction of mean eGFR from baseline was evidenced after 14 days treatment in healthy participants. The mean baseline eGFR was 95.5, 89.5, 91.5, and 93.7 ml/min per 1.73 m^2^ in placebo, AP303 50 μg, 150 μg, and 300 μg QD groups, the mean changes and the mean percentage reductions in eGFR between baseline and day 13 (1 day before the last dose) were −6.7 (−6.7%), −2.2 (−2.3%), −6.2 (−6.5%), and −10.3 (−10.6%) ml/min per 1.73 m^2^ in placebo, AP303 50 μg, 150 μg, and 300 μg QD groups, respectively (Figure 2a). In study AP303-PK-02, the mean baseline eGFR was 121.2, 108.8, and 122.7 ml/min per 1.73 m^2^ in placebo, AP303 150 μg and 300 μg QD groups. the mean changes and the mean percentage reductions in eGFR between baseline and day 14 were 0.3 (0.3%), 0.5 (0.5%), and −6.2 (−5.0%) ml/min per 1.73 m^2^ in placebo, 150 μg and 300 μg QD groups, respectively. All the reduced eGFR returned to baseline level at the 14-day off-treatment follow-up visit.

In patients with DKD and renal impairment (AP303-PK-03), the mean baseline eGFR was 45.2 and 49.1 ml/min per 1.73 m^2^ in deutaleglitazar 150 μg and in placebo group. The mean maximal change in eGFR from baseline was −4.1 (−8.3%) ml/min per 1.73 m^2^ in the deutaleglitazar group and +3.3 (+6.4%) ml/min per 1.73 m^2^ in placebo group. Detailed results are summarized in Figure 2b. A total of 4 patients who had eGFR reduction ≥ 20% within the 14-day treatment period were all in deutaleglitazar group, and the reduced eGFR returned toward the baseline after treatment discontinuation (Figure 2c).

#### Metabolic Profile

The changes from baseline in fasting glucose and lipid profile including TC, TG, HDL-C, and LDL-C were the metabolic parameters evaluated in these studies. Because both AP303-PK-01 and AP303-PK-02 studies enrolled healthy participants, the data were pooled for the presentation.

#### Lipid Profile

**Figure 3:**
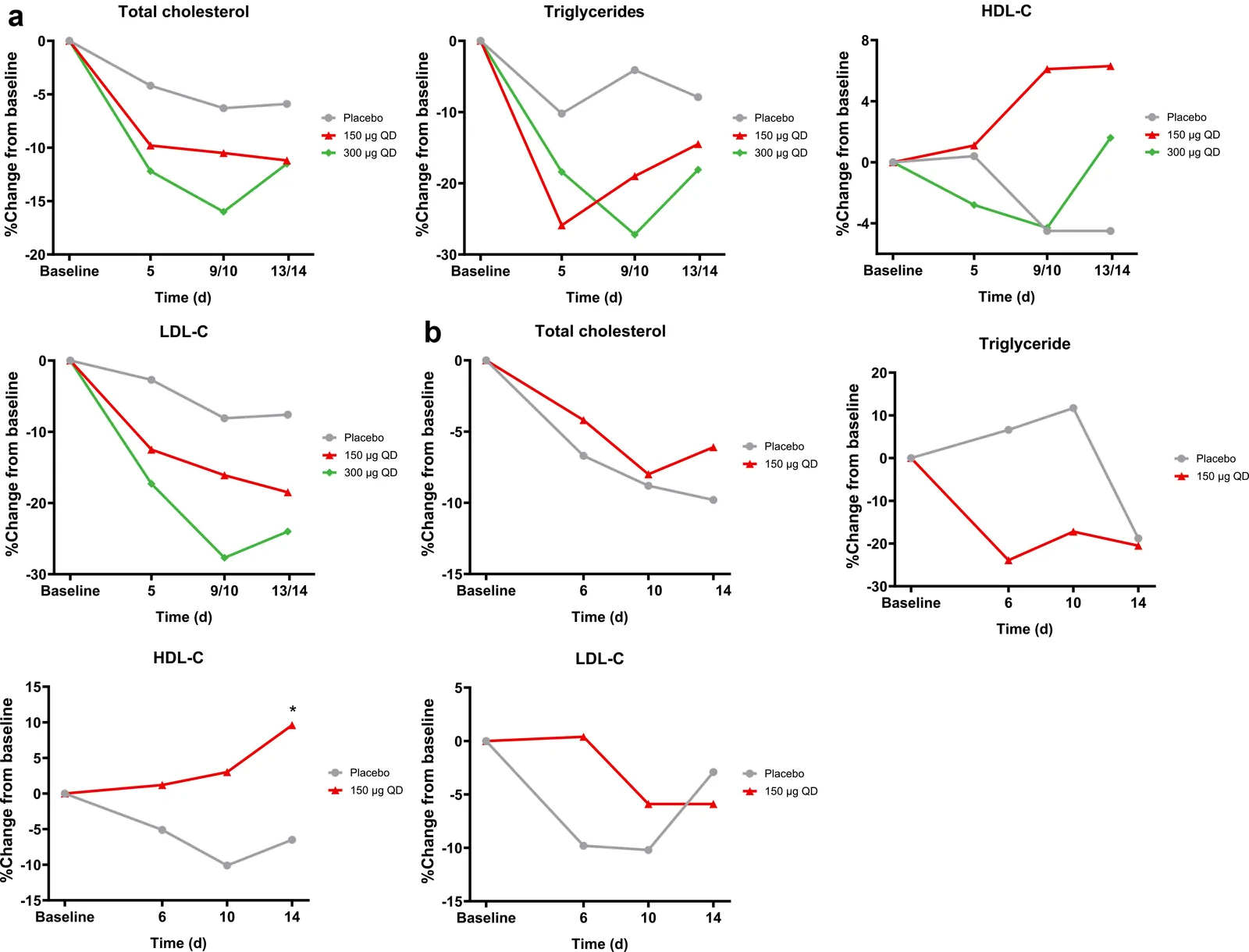
The effect of AP303 on lipid profile change in healthy participants and in patients with DKD. (a) Mean changes from baseline in lipid profile in healthy participants with normal kidney function (pooled data from AP303-PK-01 study and AP303-PK-02 study); (b) Mean changes from baseline in lipid profile in patients with DKD and eGFR 30–60 ml/min/1.73 m^2^ (data from AP303-PK-03 study); *∗P* < 0.05. eGFR, estimated glomerular filtration rate; QD, once daily.

In healthy participants, the percentage of change from baseline was −5.9%, −11.2%, −11.5% for TC; −7.9%, −14.5%, −18.1% for TG; −7.6%, −18.5%, −24.0% for LDL-C; −4.5%, 6.3%, 1.6% for HDL-C in the placebo, AP303 150 μg and 300 μg groups at the end of treatment. There was a trend of dose related decrease in TC, TG, and LDL-C, and increase in HDL-C in general (Figure 3a).

In patients with DKD, a trend of decreasing TG and increasing HDL-C was observed, but there was no clear separation between placebo (−18.8%) and deutaleglitazar (−20.5%) for TG at the end of 14-day treatment; however, the signal of increase in HDL-C was clear. At the end of 14-day treatment, HDL-C concentration showed a mean increase of 9.6% in the deutaleglitazar group, compared with a decrease of 6.5% in the placebo group (*P* = 0.031) (Figure 3b).

#### Fasting Glucose

**Figure 4:**
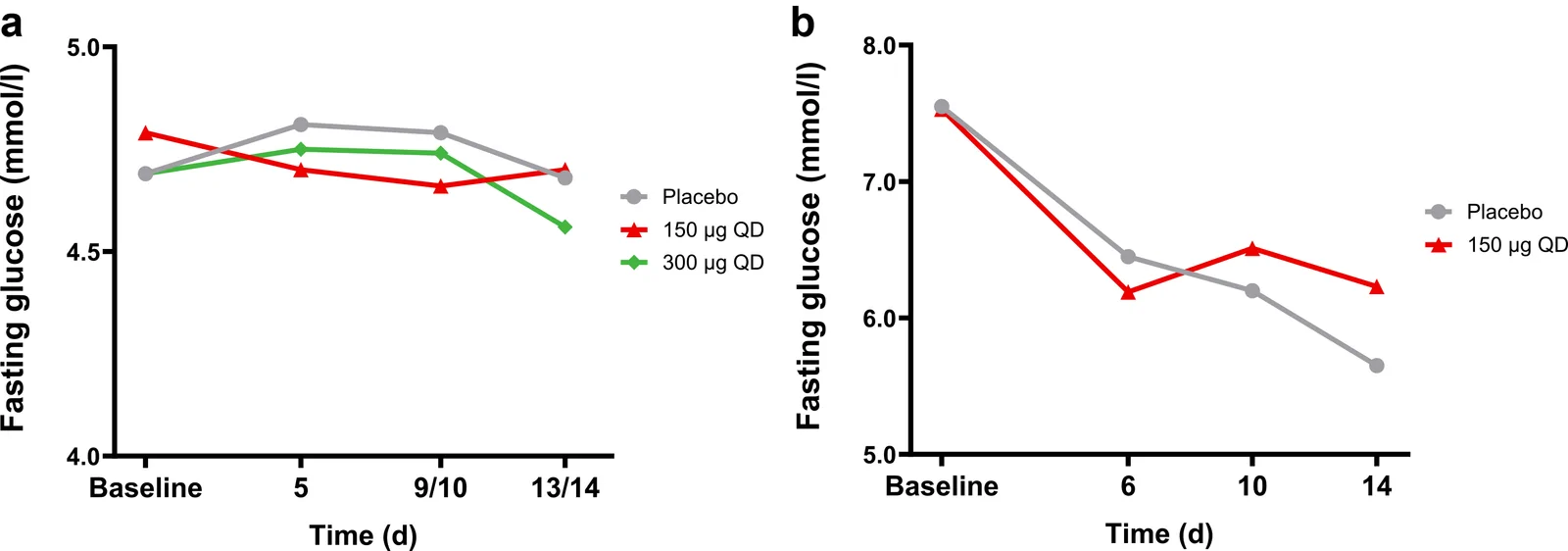
The effect of AP303 on fasting glucose change in healthy participants and in patients with DKD. (a) Fasting glucose in healthy participants with normal kidney function (pooled data from AP303-PK-01 study and AP303-PK-02 study); (b) Fasting glucose in patients with DKD and eGFR 30–60 ml/min per 1.73 m^2^ (data from AP303-PK-03 study). QD, once daily.

In healthy participants, the baseline serum fasting glucose was in the normal range, there was no significant change in fasting glucose while receiving deutaleglitazar in placebo (0.4%), AP303 150 μg (−1.9%), and 300 μg QD (−2.4%) for 14 days (Figure 4a). No hypoglycemia was observed.

In patients with DKD, the mean change in fasting glucose was 1.35 mmol/l (*n* = 10), from a baseline of 7.53 mmol/l (*n* = 11) to 6.23 mmol/l (*n* = 10) at the end of 14-day treatment in deutaleglitazar 150 μg QD group (Figure 4b). Interestingly, the placebo group also showed a reduction in glucose, the mean change in fasting glucose was 1.9 mmol/l, from baseline of 7.55 mmol/l to 5.65 mmol/l during 14-day treatment (Figure 4b).

#### UACR

Single spot UACR was only collected 2 times at screening and at the end of 14-day treatment in study AP303-PK-03. The mean baseline UACR was 1185.6 and 1328.1 mg/g, and the reduction of UACR from baseline to the end of treatment was not different (−42.2% and −64.2%) in deutaleglitazar and placebo group, respectively.

### Safety

The treatment emerged adverse events among healthy participants who received at least 1 dose of the study drugs were balanced between deutaleglitazar and placebo groups, but the treatment emerged adverse events was slightly higher in patients with DKD treated with deutaleglitazar, which was primarily attributed to the increased serum creatinine (Table 4). Except for neutropenia, no dose relationship was observed with these adverse events. Overall, 3 participants (1 in PK-01 study and 2 in PK-02 study) reported neutropenia, which occurred as early as day 5, and all of these were in the deutaleglitazar 300 μg QD groups. All the neutropenia cases were mild, and the count of participants’ neutrophil returned to normal when the study drug was discontinued without any other abnormalities.

**Table 4:** Most frequently reported treatment emerged adverse events by PT occurred in ≥ 5% Participants

Preferred term	AP303-PK-03 (Patients with DKD and eGFR 30–60 ml/min per 1.73 m^2^)		AP303-PK-01&02 (healthy participants)	
	AP303 (*N* = 12)	Placebo (*N* = 6)	AP303 (*N* = 60)	Placebo (*N* = 20)
Blood creatinine increased	5/12 (41.7%)	0	0	0
Hyperuricemia	4/12 (33.3%)	1/6 (16.7%)	0	0
Hyperkalemia	2/12 (16.7%)	0	0	0
Hypoglycemia	2/12 (16.7%)	0	0	0
Conjunctivitis/ Conjunctival hemorrhage	1/12 (8.3%)	1/6 (16.7%)	0	0
Hypertriglyceridemia	0	1/6 (16.7%)	0	0
Blood creatine phosphokinase increased	1/12 (8.3%)	0	0	1/20 (5.0%)
Eczema	1/12 (8.3%)	0	0	0
Lip infection	1/12 (8.3%)	0	0	0
Pain	1/12 (8.3%)	0	0	0
Weight increased	1/12 (8.3%)	0	0	0
Headache	0	0	5/60 (8.3%)	1/20 (5.0%)
Neutrophil decreased	0	0	3/60 (5.0%)	0
Abdominal pain	0	0	3/60 (5.0%)	0
Chest pain	0	0	0	1/20 (5.0%)
Dizziness	0	0	0	1/20 (5.0%)
Dysmenorrhea	0	0	2/60 (3.3%)	1/20 (5.0%)
Pruritus	0	0	0	1/20 (5.0%)
Rash	0	0	0	1/20 (5.0%)
Viral upper respiratory infection	0	0	0	1/20 (5.0%)
Urine WBC positive	0	0	0	1/20 (5.0%)

Only 1 participant from AP303-PK-01 study SAD cohort experienced 2 serious adverse events i.e., psychotic disorder and depressive symptoms 12 days after a single 150 μg dose of deutaleglitazar; and 1 patient from AP303-PK-03 study reported a serious adverse event of pain (common terminology criteria for adverse events grade 1) around 9 hours after receiving the first dose of 150 μg deutaleglitazar, cardiovascular or cerebrovascular event was ruled out.

The multiple doses of 150 μg deutaleglitazar were associated with mild reductions of blood pressure from baseline in patients with DKD, but no significant difference in comparison with placebo. There was no apparent effect on heart rate, QT interval, or other EKG parameters at any dose. No body weight changes from baseline, no edema or the change in hemoglobin from baseline were observed during 14 days of multiple doses in all 3 studies. Detailed results are presented in Table 5.

**Table 5:** Mean ± SD changes from baseline safety parameters after 14-day treatment in patients with DKD and renal impairment (AP303-PK-03 study)

Safety parameter	AP303 (*N* = 12)			Placebo (*N* = 6)		
	Baseline (*n* = 12)	End of treatment (Day 14) (*n* = 10)^a^	Difference (*n* = 10)	Baseline (*n* = 6)	End of treatment (Day 14) (*n* = 6)	Difference (*n* = 6)
Body weight (kg)	77.53 ± 10.44	78.14 ± 10.34	−0.94 ± 1.27	68.92 ± 9.73	67.65 ± 9.05	−1.27 ± 0.91
Systemic BP (mmHg)	140.8 ± 14.32	128.8 ± 17.18	−11.7 ± 12.46	135.2 ± 10.85	118.3 ± 6.80	−16.8 ± 5.19
Diastolic BP (mmHg)	82.8 ± 7.31	77.1 ± 8.76	−6.2 ± 6.39	82.2 ± 11.92	76.5 ± 7.50	−5.7 ± 5.32
Hemoglobin (g/l)	131.1 ± 17.40	128.0 ± 15.06	−1.9 ± 5.70	137.0 ± 16.44	132.3 ± 19.04	−4.7 ± 4.84
Hematocrit (%)	39.54 ± 5.42	38.44 ± 4.58	−0.88 ± 2.12	41.15 ± 4.39	39.18 ± 5.01	−1.97 ± 1.01
WBC (x10^9^/l)	6.81 ± 1.89	6.17 ± 1.76	−0.91 ± 0.77	6.80 ± 1.14	5.92 ± 1.15	−0.88 ± 0.89
Neutrophil (x10^9^/l)	4.55 ± 1.52	3.71 ± 1.30	−1.05 ± 0.63	4.69 ± 0.64	3.62 ± 1.04	−1.07 ± 0.79
AST (U/l)	18.2 ± 3.19	19.5 ± 4.70	1.1 ± 6.12	21.7 ± 8.48	19.8 ± 9.30	−1.8 ± 2.23
ALT (U/l)	15.6 ± 4.48	14.7 ± 4.67	−1.5 ± 6.47	19.2 ± 11.74	18.7 ± 18.00	−0.5 ± 8.07
CK (U/l)	110.8 ± 53.28	83.7 ± 30.61	−31.1 ± 53.21	126.2 ± 75.04	71.7 ± 25.34	−54.5 ± 61.42

ALT, alanine aminotransferase; AST, aspartate aminotransferase; BP, blood pressure; CK, creatine kinase; WBC, white blood cell.

a 2 participants in AP303 group prematurely discontinued the treatment due to adverse events.

## Discussion

Halting the progression of DKD remains a key goal in diabetes management and may be possible if the elevated glomerular capillary pressure can be normalized to a physiologic level, podocyte depletion can be prevented, and key progression factors, such as hyperglycemia and dyslipidemia can be addressed. The novel dual PPARα/γ agonists deutaleglitazar has the potential to offer these combined clinical benefits. The presented studies not only characterized the PK profile of deutaleglitazar, but also revealed certain signals related to potential renal protection.

A dose related decline in eGFR was observed from day 5 to the end of 14 days treatment of deutaleglitazar in heathy participants, which returned toward to the baseline level 14 days after the last dose. The reduction and reversibility of eGFR were also observed in patients with DKD and renal impairment. No clinically significant abnormality was observed in urinalyses including cellularity or sediment. This is a likely hemodynamic effect that is expected on deutaleglitazar treatment. Because of the short treatment duration in the current studies, it may not have reached a nadir or stable level or may need a longer time to recover after participants discontinued the drug. Deutaleglitazar is the deuterated form of aleglitazar. In a similar population to the AP303-PK-03 study, aleglitazar treatment resulted in a ∼8% mean eGFR reduction at week 2 which was similar to the mean eGFR reduction in our AP303-PK-03 study after 14 days treatment at the same dose level of 150 μg QD, but the eGFR reduction associated with aleglitazar reached a plateau around week 8 with a 15% mean decrease in eGFR from baseline and was not progressive out to week 52, the decreased eGFR was reversible 8 weeks off treatment.^12^ This glomerular hemodynamic change is likely due to the PPARα effect of these dual PPARα/γ agonists (e.g., aleglitaar) and it is the true GFR changes measured by iohexol.^13^ Certain PPARα agonists are CYP4A inducers that increase 20-HETE which decreases eGFR by preglomerular myogenic constriction.^14^, ^15^, ^16^ In addition, in the same aleglitazar study in patients with macroalbuminuria, UACR decreased from baseline to the end of 52-week treatment by 59.3% with aleglitazar as well as by 50.5% with pioglitazone.^12^ It is known that PPARγ agonist inhibits heparinase expression in both endothelial cells and podocytes, prevents podocyte foot effacement, reduces urinary podocyte, and decreases trans-endothelial albumin passage.^11^^,^^17^ Single spot UACR was only collected 2 times at screening and at the end of 14-day treatment in study AP303-PK-03. There was no difference in UACR changes from baseline between deutaleglitazar and placebo groups. Duplicate UACR collections at multiple-time points or 24-hour UACR change was not collected as 1 of the pharmacodynamic end points, which was a limitation of current study design. However, in a head-to-head comparison study between deutaleglitazar and pioglitazone conducted in an animal model of DKD (BLKS db/db mice with unilateral nephrectomy), over 50% reduction in 24-hour urinary albumin excretion with improved renal pathologic changes were observed in both deutaleglitazar and pioglitazone groups at week 6 and week 10 (unpublished data).

Low level of HDL-C is a well-established biomarker for higher cardiovascular risk especially in patients with diabetic dyslipidemia, however, not all the products raising HDL-C decrease cardiovascular risk with the exception of a PPARα agonist.^18^ Interestingly, subsequent analyses of all major PPARα trials showed consistently better cardiovascular outcomes in the patient subgroups with mixed dyslipidemia (low HDL-C and high TG), characteristically seen in patients with T2D.^19^ A healthy lipid profile was observed early in the course of the therapy, deutaleglitazar showed a signal of HDL-C increase in the presented studies, resulting from its PPARα effect. In addition, PPARγ agonists also affect cardiovascular outcomes, but this effect is controversial,^20^ and it is compound specific. Pioglitazone could improve cardiovascular outcome in T2D who are at high cardiovascular risk,^21^ especially in diabetes with chronic kidney disease.^22^ Deutaleglitazar exerts PPARγ activity in reducing fasting glucose by increasing insulin sensitivity. Like other PPARγ agonist, deutaleglitazar monotherapy has not been associated with hypoglycemia, but it increases the propensity for hypoglycemia when used in conjunction with insulin or sulfonylureas. Only 2 patients with DKD in AP303-PK-03 study adjusted their dose of concomitant use of insulin to avoid hypoglycemia were in deutaleglitazar group. However, reduction of fasting glucose was also observed in the placebo group in AP303-PK-03 study. Because all patients entering the study receiving at least 1 glycose lowering products in both treatment groups, improved diet with lifestyle changes (staying in the clinical research unit for 14 days), may result in a nonsignificant difference between placebo and deutaleglitazar groups. It looks like that both PPARα- and PPARγ-related effects were observed under the deutaleglitazar 150 μg QD dosing regimen, and the magnitude of the changes in eGFR, HDL-C, and the fasting glucose were in the therapeutic target ranges, which supports the notion that deutaleglitazar has a balanced binding affinity toward both the PPARα and PPARγ receptor subtypes.

Exposure to deutaleglitazar increased in a dose-proportional manner across the dose escalation study, following both single doses (50 μg, 150 μg, 300 μg, or 600 μg) and multiple doses (50 μg, 150 μg, or 300 μg QD for 14 days). Peak plasma concentrations reached at approximately 2 hours after oral administration under fasted conditions, and at approximately 2.2 hours after administration of 150 μg of deutaleglitazar with food. Except for a slight delay in T_max_ and a slight decrease in C_max_, food had no clinically relevant effect on the bioavailability of deutaleglitazar. The apparent terminal half-life of deutaleglitazar was in the range of 8 to 11 hours. There was no accumulation of deutaleglitazar with QD dosing regimen. A total of 2 metabolites, M1 and M2, were confirmed as the major circulating metabolites, and both are pharmacologically inactive. Renal excretion of the parent drug was negligible, which is consistent with its known liver metabolic pathway. In patients with DKD and renal impairment (eGFR 30–60 ml/min per 1.73 m^2^) receiving deutaleglitazar 150 μg QD, there was a slight increase in AUC_0-tau_ (by 16.7%), these findings are not considered to be clinically significant.

Based on the pharmacologic mechanism of action, the observed safety profile of deutaleglitazar was consistent with the combined pattern of adverse events from the marketed PPARα (fenofibrate and gemfibrozil) as well as PPARγ agonist (pioglitazone). Neutropenia was observed in PPARα agonist (gemfibrozil) and dual PPAR-α/γ agonist (aleglitazar) treatment in the high dose group,^23^ which could be dose dependent and related to PPAR-α activation.^24^^,^^25^ Neutropenia was not observed in the dose of deutaleglitazar 150 μg QD that would be the target dose in phase 2 and 3 studies. Increase in serum creatinine or decrease in GFR was the effect of PPARα activation that was observed in fenofibrate and in dual PPARα/γ agonist aleglitazar and Tesaglitazar. Progressive decrease in eGFR resulted in the discontinuation of tesaglitazar development, which may be secondary to its unique elimination pathway.^26^ Unlike tesaglitazar, both aleglitazar and deutaleglitazar are not metabolized through kidney, they had clear hemodynamic effect and reversibility of eGFR after stopping treatment. Previous studies revealed that an early drop of eGFR followed by stabilization may be an efficacy signal for renal protection in long run.^27^ The major adverse effects of PPARγ agonist include gain in body weight and fluid retention. The decrease in hemoglobin concentration that along with edema is an indicator of fluid retention, which was observed in both pioglitazone and aleglitazar studies.^28^ Deutaleglitazar has been developed for an optimized PPAR α/γ activity with a relatively weaker PPAR γ activity to improve its safety margin in comparison to aleglitazar. No body weight gain, edema, or the drop of hemoglobin were observed in our studies, which could be because of the short treatment duration or the weaker PPARγ effect.

In conclusion, the PK profile of duetaleglitazar supports its administration orally once a day with or without meals. The minor differences of PK profile in Caucasian versus Asian, and in normal kidney function versus in renal impairment are not considered to be clinically significant in respect to future clinical trials with duetaleglitazar and therefore no different dosing regimen is suggested. The results from these studies suggested that duetaleglitazar combines the desirable efficacy signal of both PPARα agonists (regulating glomerular pressure plus improvement of lipid profile) and PPARγ agonist (improvement of glycemic control) as well as having a manageable safety profile. A phase 2 study is planned to evaluate albuminuria reduction as well as the acute and chronic eGFR slope changes in patients with DKD and macroalbuminuria.

## Disclosure

VP has received honoraria for steering committee, data monitoring committee, or advisory board roles or for scientific presentations from AstraZeneca, Bayer, Boehringer Ingelheim, Chinook, GlaxoSmithKline, Janssen, Novartis, Novo Nordisk, Otsuka, Travere, Tricida, and UptoDate; and is a Board Director for George Clinical, St. Vincents Health Australia, and several independent medical research institutes.

HZ has received consultancy for steering committee roles from Calliditas, Chinook, Otsuka, Novartis, Roche, Vera, Alexion/AstraZeneca, Alpine/Vertex, George Clinical, Biogen, and Takeda; she is a member of RESOLVE Data Safety Monitoring Committee.

JCL received grants from the China National Funds for distinguished young Scientists and Capital’s funds for Health Improvement and Research (grant 2024-1-4073) during the conduct of the study, and consulting fees and support for attending meetings and/or travel from Otsuka, Novartis, Everest Medicines and AstraZeneca Pharmaceuticals outside the submitted work. SLF, YG, and HYL have no conflict of interest to declare. YMY, YRZ, CL, HDZ, and JT are employees of Alebund Pharmaceuticals (Shanghai) Ltd.

## Supplementary Material

Supplementary File (PDF) CONSORT Checklist.
